# Descriptors for dielectric constants of perovskite-type oxides by materials informatics with first-principles density functional theory

**DOI:** 10.1080/14686996.2020.1724824

**Published:** 2020-02-25

**Authors:** Yusuke Noda, Masanari Otake, Masanobu Nakayama

**Affiliations:** a Center for Materials Research by Information Integration (CMI^2^), Research and Services Division of Materials Data and Integrated System (MaDIS), National Institute for Materials Science (NIMS), Tsukuba, Japan; b Frontier Research Institute for Materials Science (FRIMS), Nagoya Institute of Technology, Nagoya, Japan; c Global Research Center for Environment and Energy Based on Nanomaterials Science (GREEN), National Institute for Materials Science (NIMS), Tsukuba, Japan; d Elements Strategy Initiative for Catalysts and Batteries (ESICB), Kyoto University, Kyoto, Japan

**Keywords:** Perovskite-type oxides, partial least-squares regression analysis, first-principles calculations, materials informatics, dielectric materials, 202 Dielectrics / Piezoelectrics / Insulators, 401 1st principle calculations, 404 Materials informatics / Genomics

## Abstract

Dielectric materials that can realize downsizing and higher performance in electric devices are in demand. Perovskite-type materials of the form ABO_3_ are potential candidates. However, because of the numerous conceivable compositions of perovskite-type oxides, finding the best composition is technically difficult. To obtain a reasonable guideline for material design, we aim to clarify the relationship between the dielectric constants and other physical and chemical properties of perovskite-type oxides using first-principles density functional theory (DFT) and partial least-squares regression analysis. The more important factors affecting the dielectric constants are predicted based on variable importance in projection (VIP) scores. The dielectric constant strongly correlates with the ionicity of the B cations and the density of states of the conduction bands of the B cations.

## GRAPHICAL ABSTRACT

**Figure:**
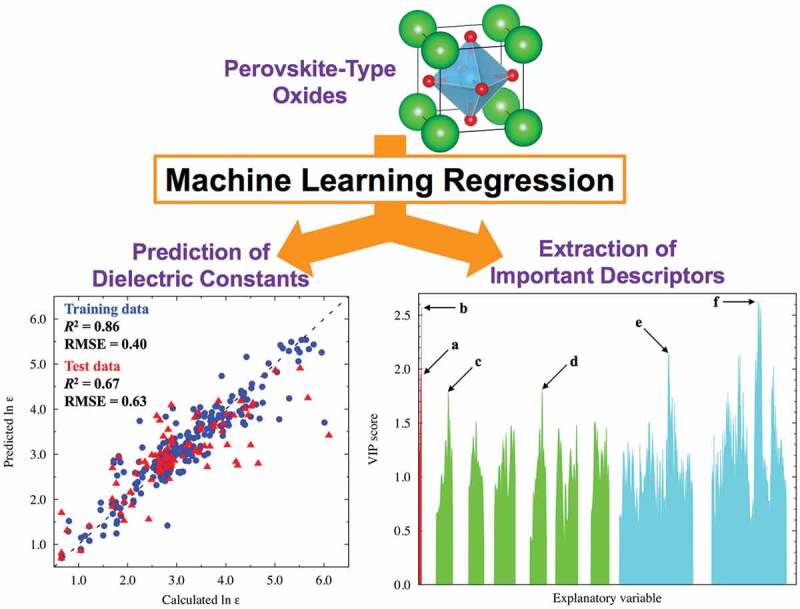

## 1. Introduction

In recent years, increasing attention has been paid toward simulating informatics-aided materials. One of the aims is to discover novel functional materials by combining data scientific methods and computational material simulations [1]. Some research groups have been successful in efficiently discovering novel functional materials using materials informatics techniques, such as high-throughput screening and machine learning methods, in various areas of materials science [2–9]. Materials informatics can also substantially contribute to data mining by clarifying the relationships, i.e. the so-called quantitative structure-property relationships (QSPRs), between the structures and properties of functional materials [10]. In this case, regression analysis methods, such as partial least-squares (PLS) regression, principal component analysis (PCA), support vector regression (SVR), and least absolute shrinkage and selection operator (LASSO) regression, are often used for building regression models for molecules and crystal structures [11–19]. Clarifying the descriptors indicating the QSPRs will accelerate the design of functional materials.

Broderick et al. conducted successive studies on mining descriptors [20–23]. They clarified the relationship between the electronic states and physical properties of target materials through a data mining analysis, with the density of states (DOS) as spectral data. Using a combination analysis with PCA and PLS methods, they found a strong correlation between several characteristic DOS peaks and bulk moduli for binary alloys [23], which helped in understanding the important factors affecting a particular property and in predicting the property of novel functional materials without serious evaluations (e.g. experimental measurements or expensive theoretical simulations such as first-principles calculations).

In this paper, we introduce a PLS regression analysis to reveal the correlations between dielectric properties and different physical and chemical properties of perovskite-type materials with the general formula ABO_3_. Some of the perovskite-type oxides are well-known dielectric materials [24–27]. In particular, barium titanate BaTiO_3_ and strontium titanate SrTiO_3_ are commercially available ferroelectric materials. The rest of this paper is organized as follows. In section 2, we show the methodology of the first-principles calculations based on the density functional theory (DFT) [28] and the procedure to perform the PLS regression analysis [29–31]. In section 3, we show the suitable descriptors used to predict the dielectric properties of target materials and the accuracy with which they can be predicted. Finally, the conclusions drawn from this study are given in section 4.

## 2. Computational details

### 2.1. First-principles calculation

We used the Vienna *ab initio* simulation package (VASP) [32–35] based on the DFT with a projector augmented-wave method [36,37] and a plane-wave basis set to optimize the unit cells and atomic coordinates of the target materials. A Perdew–Burke–Ernzerhof generalized gradient approximation (GGA-type) exchange-correlation functional modified for solid materials [38] was used. The cutoff energy for the plane-wave basis was set to 500 eV. The unit cells of all the structures were optimized with the ***k***-point resolution set to 1,000 for a high-throughput investigation (i.e. *N_x_, N_y_*, and *N_z_*, which are the numbers of grids in the *k_x_-, k_y_*-, and *k_z_*-directions of the reciprocal space, respectively, were set to satisfy the condition *N_x_* × *N_y_* × *N_z_* × *N*_atom_ ≈ 1,000, where *N*_atom_ is the number of atoms in each structure). The following materials were considered in this study. The composition was ABO_3_, with the cation A being Mg^2+^, Ca^2+^, Sr^2+^, Ba^2+^, or Zn^2+^ and cation B being Ti^4+^, Zr^4+^, Hf^4+^, Si^4+^, Ge^4+^, or Sn^4+^ (the number of compositions was 30). In addition, 12 space group symmetries of the target materials were selected: Pnma (#62), Fmmm (#69), Imma (#74), P4mm (#99), P4/mmm (#123), R3 (#148), R3m (#160), R3c (#161), R3m (#166), R3c (#167), P6_3_/mmc (#194), and Pm3m (#221). Hence, 360 perovskite-type oxides were prepared as sample data for the PLS regression analysis.

### 2.2. PLS regression analysis

The following descriptors were used as explanatory variables in the PLS regression analysis: the cohesive energies and bulk moduli of the target materials; Pauling electronegativities [39,40], Shannon ionic radii [41], differences between the Bader charges [42] and the formal charges (separate values are given for cations A and B), and DOS spectral data for both A and B cations; bond length of A − O and B − O; and radial distribution function (RDF) spectral data of A − A, A − B, A − O, B − B, B − O, and O − O combinations. The cohesive energies (differences between their total energies and the energy of an isolated atom for each element) were calculated beforehand. The bulk moduli were estimated by fitting the Murnaghan equation of state to an energy–volume curve [43]:

(1) $$\begin{array}{rl} & E\left(V\right)=E_{0}+B_{0}V_{0}\left[\frac{1}{B_{0}^{\prime }\left(B_{0}^{\prime }-1\right)}{\left(\frac{V}{V_{0}}\right)}^{\left(B_{0}^{\prime }-1\right)}\right.\\ & \;\;\;\;\;\;\;\;\;\;\;\;\;\;\;\;\;\;\;\;\;\left.\;+\frac{1}{B_{0}^{\prime }}\frac{V}{V_{0}}-\frac{1}{B_{0}^{\prime }-1}\right] \end{array}$$

where *E*_0_ and *V*_0_ are the equilibrium total energy and volume under zero pressure conditions, respectively, and *B*_0_ and *B*ʹ_0_ are the equilibrium bulk modulus and its first derivative with respect to the pressure, respectively. The DOS spectra of the A and B cations were determined in the −14.9 to 14.9 eV energy range. The grid size for the DOS was 0.1 eV (i.e. the dimension was 299 for each DOS spectrum). The RDF spectra of the elemental combinations were also calculated with a grid size of 0.045 Å and a maximum distance of 4.5 Å (i.e. the dimension was 100 for each RDF spectrum); the full width of Gaussian broadening was 0.2 eV. Eventually, there were 1,208 dimensions for the explanatory variables in total in the PLS regression model. To evaluate the dielectric constants of the perovskite-type oxides, we used density functional perturbation theory (DFPT) [44,45] implemented in the VASP code. The dielectric constant *ε* is the average of the diagonal components *ε_ii_* (*i* = 1, 2, and 3) of the dielectric tensor:

(2) $$\varepsilon =\;(\varepsilon_{11}+\varepsilon_{22}+\varepsilon_{33})/3$$

In PLS regression analysis, we estimated an index to determine the superiority of the regression coefficients. Wold et al. proposed variable importance in projection (VIP) scores, which reflect the influence of the explanatory variables on the PLS regression model [46,47]. Explanatory variables with large VIP scores are important for building the PLS regression model. We used JMP® software [48,49] and carried out the PLS regression analysis to extract suitable descriptors for predicting the dielectric constants of the perovskite-type oxides.

## 3. Results and discussion

**Figure 1.**
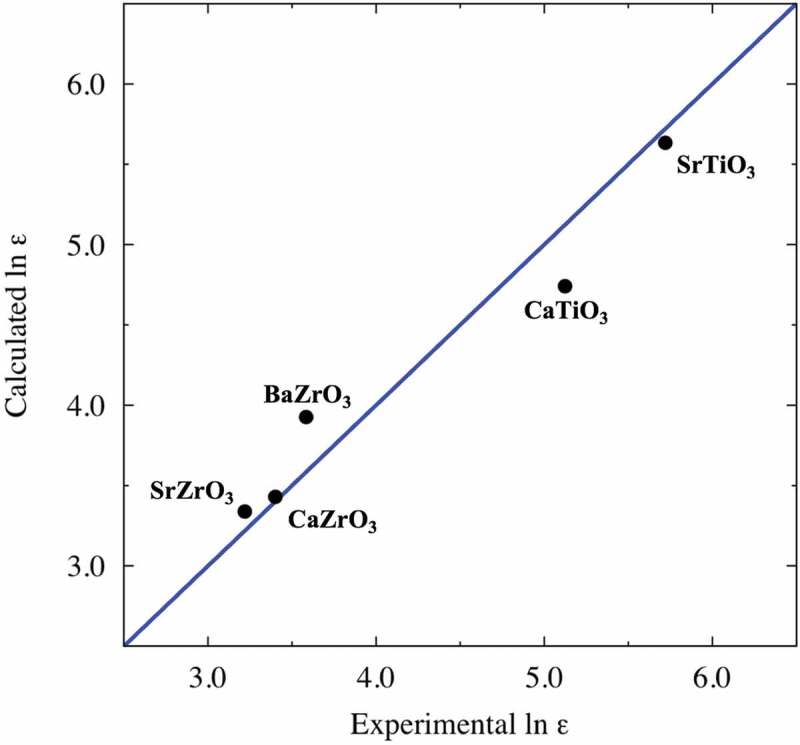
Comparing calculated and experimental results of logarithmic dielectric constant, ln *ε*. The blue line is an ideal straight line, indicating that the calculated value is equal to the experimental value. Experimental results are obtained from Refs 50 − 53

We first present the results of the dielectric constants of the perovskite-type oxides obtained using the first-principles calculations with the DFPT. The resulting dielectric constants of five perovskite-type compounds, namely CaTiO_3_ (Pnma) [50], CaZrO_3_ (Pnma) [51], SrTiO_3_ (Pm3m) [52], SrZrO_3_ (Pnma) [53], and BaZrO_3_ (Pm3m) [51] are compared with the experimental results measured nearly at zero Kelvin, as shown in Figure 1. In fact, some differences are observed between the theoretical and experimental results in this study. It is well-known that DFT with GGA-type functionals calculates larger dielectric constants because GGA-type functionals overestimate the lattice constants of crystal structures, which affects low-frequency phonon modes [54,55]. Table 1 lists some of the highest calculated values of the dielectric constants in our DFPT simulation. We find that SrTiO_3_-based materials exhibit relatively high dielectric constants, and some of their prediction results are close to their calculation results. Moreover, as a tendency of the optimized structures of the perovskite-type oxides, almost all of the targets, except structures with the space group symmetries R$\bar{3}$ and P6_3_/mmc, exhibit similar RDF profiles to a cubic system (Pm$\bar{3}$m) or closely resemble them (for example, some structures with Pnma and P4mm symmetries transform into Pm3m-like systems due through structural optimization). We also confirmed no change in space group before and after structural relaxation using the FINDSYM program [56]. However, we found differences in the dielectric constants even for the same composition because the dielectric constants obtained using the DFPT calculations are sensitive to slight differences in the atomic coordinates. Note that BO_6_ octahedra link through their vertices in regular perovskites, such as those of Pm$\bar{3}$m, and P4mm symmetry, while perovskites with R and P6_3_/mmc symmetries have edge- or face-sharing BO_6_ octahedra.

**Table 1.** Highest values of dielectric constants of perovskite-type oxides estimated from DFPT. Dielectric constant values predicted using PLS and error values are also shown in the table. Absolute values of error over 1.0 are highlighted in bold

Composition	Space group	Calculated ln *ε*	Predicted ln *ε*	Error *Δ*(ln *ε*)
SrZrO_3_	P4mm	6.10	3.41	**−2.69**
SrHfO_3_	R3m	6.01	3.70	**−2.31**
SrTiO_3_	R3m	5.95	5.26	−0.69
SrTiO_3_	R3c	5.69	5.33	−0.36
CaTiO_3_	P6_3_/mmc	5.67	4.25	**−1.42**
SrTiO_3_	Pm3m	5.63	5.54	−0.09
SrTiO_3_	P4/mmm	5.54	5.53	−0.01
SrTiO_3_	Fmmm	5.50	5.26	−0.24
SrTiO_3_	Imma	5.48	5.06	−0.42
SrTiO_3_	R3m	5.46	5.33	−0.13
SrTiO_3_	Pnma	5.43	5.28	−0.15
SrTiO_3_	P4mm	5.33	5.49	0.16
SrHfO_3_	P6_3_/mmc	5.29	4.24	**−1.05**
SrTiO_3_	R3c	5.28	5.29	0.01
ZnTiO_3_	Pnma	5.12	4.70	−0.42
BaTiO_3_	R3c	5.11	5.42	0.31
CaSnO_3_	Fmmm	5.09	3.78	**−1.31**
CaTiO_3_	Imma	5.04	4.82	−0.22
CaTiO_3_	Fmmm	5.01	4.86	−0.15
BaTiO_3_	P6_3_/mmc	4.87	5.01	0.14
MgTiO_3_	P4mm	4.79	4.75	−0.04
CaGeO_3_	P6_3_/mmc	4.75	4.14	−0.61
CaTiO_3_	Pnma	4.74	4.50	−0.24
SrZrO_3_	R3c	4.69	3.79	−0.90
CaSnO_3_	P6_3_/mmc	4.66	2.79	**−1.87**
BaSiO_3_	Pm3m	4.62	4.10	−0.52

**Figure 2.**
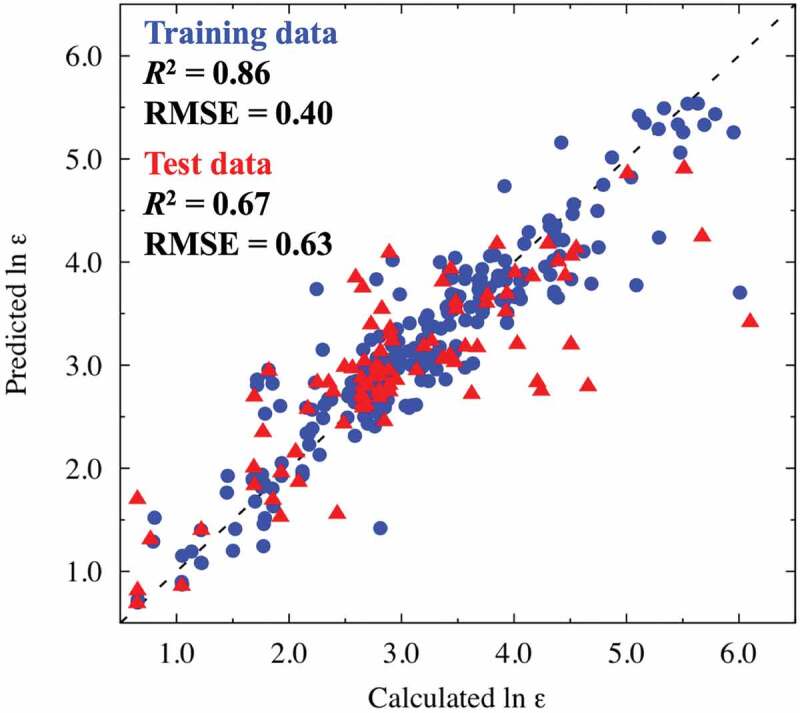
Comparisons between calculated (DFPT) and predicted (PLS) logarithmic dielectric constants ln *ε* for training data (blue circles) and test data (red triangles). The dash line is the ideal straight line where predicted data equal calculated data

We build the PLS regression model to predict DFPT results of dielectric constants of perovskite-type oxides using 66.7% of all samples as training data, and determine the number of components in the PLS regression model using the other 33.3% of samples as test data. Prediction errors against the number of components in the PLS regression model are shown in Supplemental Figure S1. Generally, the regression model with the lowest prediction error is adopted as the best prediction model; hence the PLS regression model with ten components is adopted in this study. Figure 2 shows the diagnostic plot of the logarithm of the dielectric constants (ln *ε*) between the results obtained using the DFPT and the ones predicted using the PLS regression model for training data and test data. Prediction abilities are fairly good, with coefficients of determination (*R*^2^) and root-mean-square error (RMSE) values of 0.86 and 0.40 for training data, and 0.67 and 0.63 for test data, respectively. The deviations between fitted and DFPT-derived values tend to be large in the high ln *ε* region. These deviations are ascribable to shallow potential curves around B-site cations, and the ln *ε* values of these materials are sensitive to small changes in the potential curve. We recalculated the PLS regression after removing the explanatory variables with low VIP scores (< 0.8) (*vide infra*) and confirmed no significant difference in fitting quality (RMSEs of 0.43 and 0.57 were calculated for the training and test data, respectively). In addition, we confirmed no significant change in RMSE for test datasets, even after the datasets related to R3 and P6_3_/mmc symmetries, whose structures contains face- or edge-shared BO_6_ octahedra, were removed from the PLS regression (see Supplemental Figure S2). Hereinafter, we discuss the PLS regression results presented in Figure 2.

**Figure 3.**
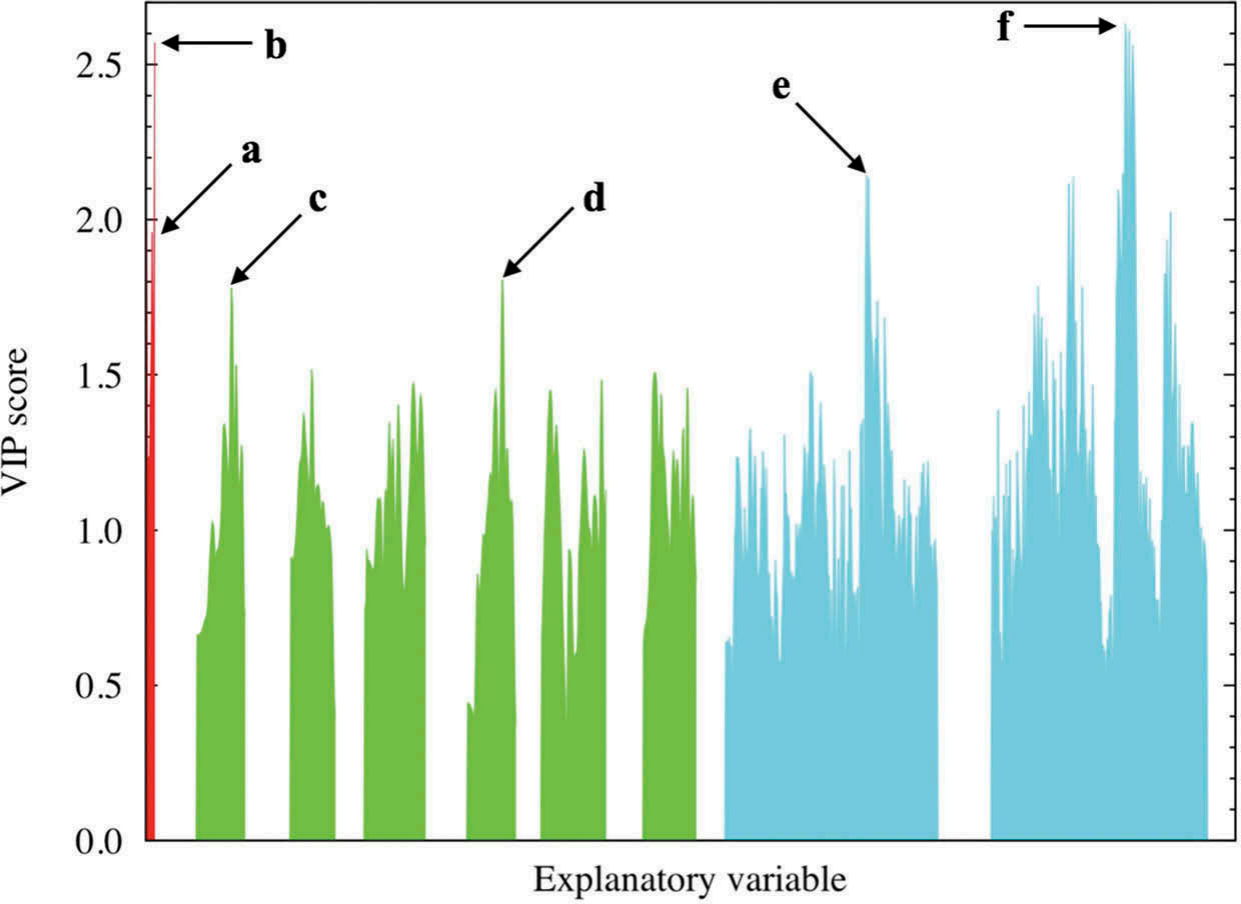
VIP scores of explanatory variables in the PLS model. The red-, green-, and cyan-colored peaks correspond to the basic physical and chemical properties (e.g. cohesive energy and ionic radii of A and B cations), RDF (A − A, A − B, A − O, B − B, B − O, and O − O combinations), and DOS (A and B cations), respectively. The arrows a − f indicate the more important explanatory variables with high VIP scores

The present descriptor sets contain important factors affecting the dielectric constants of the various perovskite-type oxides. Figure 3 shows the resulting VIP scores, which reflect the importance of each explanatory variable in fitting the explanatory and objective variables. In our PLS regression analysis, the following descriptors, which are denoted by a–f in Figure 3, for a good prediction performance of the dielectric constants are extracted: (a) ionic radii of the A cations, (b) differences between the Bader and formal charges of the B cations, (c) RDF of the A − A combination (approximately 3.8 Å), (d) RDF of the B − B combination (approximately 3.8 Å), (e) DOS of the conduction bands derived from the A cations (approximately 4.0 eV vs. the Fermi level), and (f) DOS of the conduction bands derived from the B cations (approximately 3.0 eV vs. the Fermi level). PLS regression was also carried out by adding band gap values extracted from DOS spectra as explanatory variables, since the previous paper revealed clear correlations between band gap and dielectric properties [55]. However, no significant change in fitting quality was observed. The RMSE for the test data is 0.54 when band gaps are included in datasets and 14 components are used for regression (see Supplemental Figure S3). We also note that the energy scale for DOS spectra is set to be zero for the Fermi level. Another energy reference considered to be physical reasonable is the vacuum level, although energy alignment to the vacuum level is technically difficult due to periodic boundary conditions. Instead, we aligned the energy level using the O 2s core state; however, the PLS regression results are essentially the same, irrespective of the choice of energy reference. The details are presented in Supplemental Figure S4. Hereinafter, PLS regression results using DOS energies referenced to the Fermi level are considered. We discuss the above six factors separately in the following.

**Factor (a)**: Despite the high VIP score for the ionic radii of the A cations (~1.96), there is no clear relationship with the dielectric constants, as shown in Supplemental Figure S5. The averaged dielectric constants of the compounds including the same A ions gradually increase with respect to the ionic radius, from Mg (0.72 Å) to Sr (1.18 Å); this is consistent with the positive coefficient obtained for the PLS-derived prediction function. However, the change is within the standard deviation range, and the averaged dielectric constant decreases, from Sr (1.18 Å) to Ba (1.35 Å). Therefore, the ionic radius of the A ions may partly affect the dielectric constants; however, the factor is not dominant, i.e. no trend is observed because of the effect of the other factors.

**Factor (b)**: The VIP score for the differences between the Bader and formal charges of the B cations is approximately 2.57. Here, we investigate the relationship between the dielectric properties and the Bader charge of the B ions. Supplemental Figure S6 shows the results. Overall, the dielectric constants tend to increase with decreasing Bader charge despite the large scattering. In detail, as listed in Table 1, some of the Ti-containing perovskite-type oxides appear as high dielectric constant materials. This is consistent with the fact that the averaged Bader charges of the Ti ions (+2.58) is the lowest compared with those of the other cations: +3.44 (Zr^4+^), +3.94 (Hf^4+^), and +4.00 (Si^4+^, Ge^4+^, and Sn^4+^). We infer that the deviation from the nominal charges is related to the covalency with the oxide ions, which induces a second-order Jahn–Teller effect (SOJT) for the *d*^0^ ions. As the SOJT significantly affects the dielectric performance, the Bader charges of the B ions lead to a high VIP score for the present PLS fitting.

**Figure 4.**
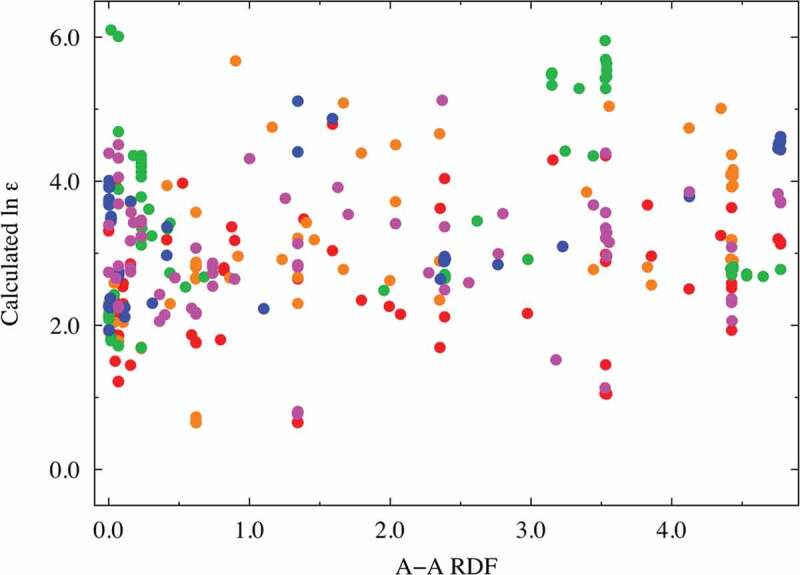
Dielectric constant plot vs. A − A RDF (in the range of 3.69–3.87 Å) explanatory variables with high VIP scores. The red, orange, green, blue, and magenta points correspond to Mg, Ca, Sr, Ba, and Zn compounds, respectively

**Figure 5.**
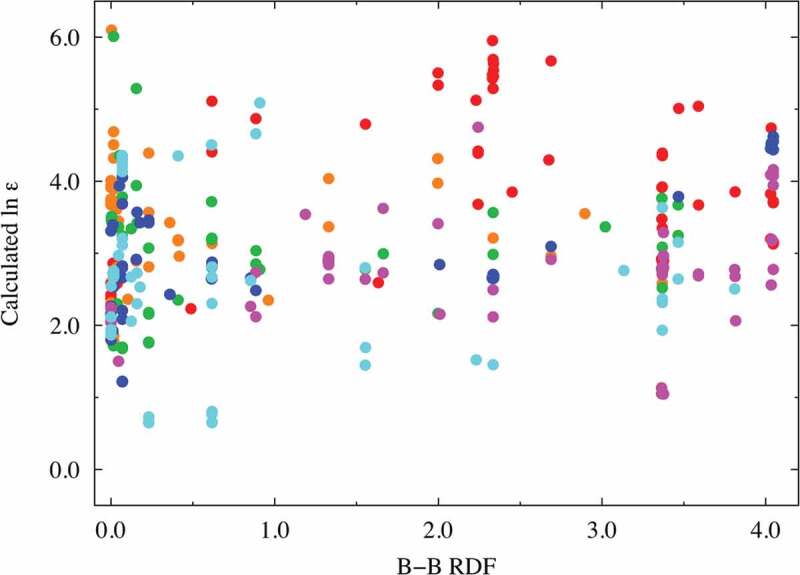
Dielectric constant plot vs. B − B RDF (in the range of 3.69–3.87 Å) explanatory variables with high VIP scores. The red, orange, green, blue, magenta, and cyan points correspond to Ti, Zr, Hf, Si, Ge, and Sn compounds, respectively

**Factors (c) and (d)**: The RDF spectral data of the A − A combinations at approximately 3.8 Å were extracted as one of the better descriptors (VIP ~ 1.78 at most) with positive coefficients. Similarly, the RDF spectral data of the B − B combinations at approximately 3.8 Å were extracted as one of the better descriptors (VIP ~ 1.81 at most) with negative coefficients. Here, we investigate the relationship between the dielectric constants and the important RDF spectral data. Figures 4 and 5 show this relationship for the A − A combinations (in the range of 3.69–3.87 Å) and B − B combinations (in the range of 3.69–3.87 Å), respectively. Despite the high VIP score for the RDF at a certain distance for the A − A and/or B − B interactions, no clear relationship is observed between the RDF values and the dielectric constants. This indicates that multiple factors affect the dielectric constants. For comparison and validation, the dielectric constant is plotted against the interatomic distance of the A − A and B − B ions, as shown in Supplemental Figures S7(a) and S7(b). Again, no clear relationship is observed between the interatomic distance and the dielectric property in both figures. This shows that the A − A and/or B − B interatomic distances are not dominant factors; however, they might be complementary factors according to the VIP analysis, as shown in Figure 3. The dielectric constants of the selected compositions, namely CaTiO_3_, SrTiO_3_, BaTiO_3_, and CaSnO_3_, are replotted as a function of the B − B interatomic distance, as shown in Supplemental Figure S7(c). In particular, the dielectric constant of CaTiO_3_ monotonically decreases with respect to the B − B interatomic distance, whereas no clear relationship is seen for BaTiO_3_. The bond length of the nearest neighbor A − A or B − B ions is partly related to the dielectric property, though this factor is not dominant.

**Figure 6.**
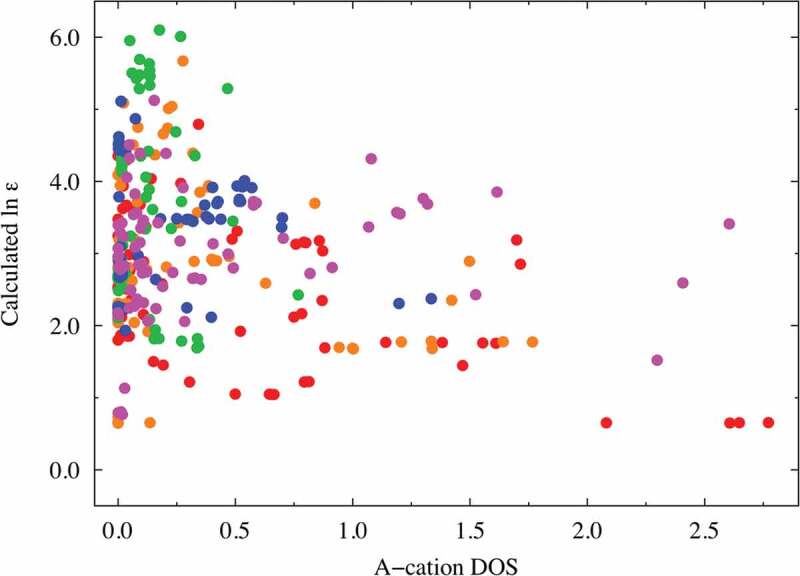
Dielectric constant plot vs. A-cation DOS (in the range of 3.8–4.4 eV) explanatory variables with high VIP scores. The red, orange, green, blue, and magenta points correspond to Mg, Ca, Sr, Ba, and Zn compounds, respectively

**Figure 7.**
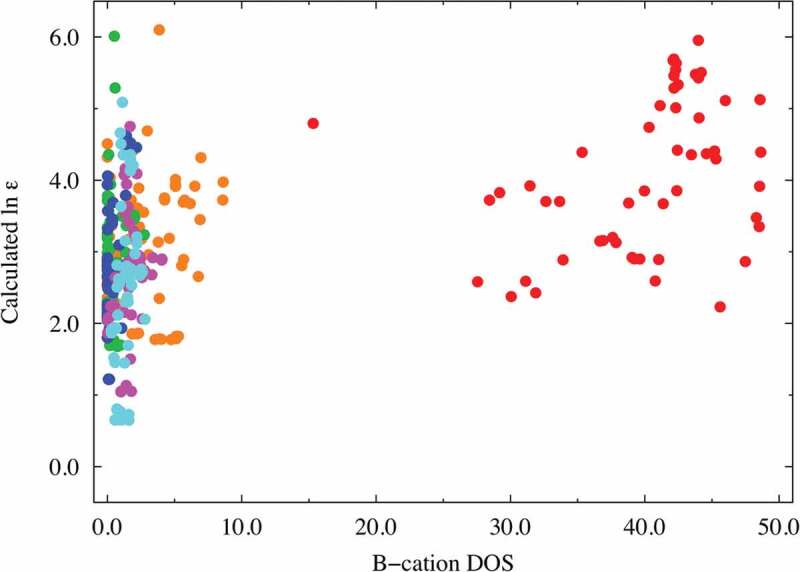
Dielectric constant plot vs. B-cation DOS (in the range of 1.9–3.9 eV) explanatory variables with high VIP scores. The red, orange, green, blue, magenta, and cyan points correspond to Ti, Zr, Hf, Si, Ge, and Sn compounds, respectively

**Factors (e) and (f)**: The DOS spectral data are more important than the RDF descriptors (factors (c) and (d)) in predicting the dielectric constants in terms of the VIP score from our PLS regression analysis. The A-cation DOS spectral data at approximately 4.0 eV were extracted as one of the better descriptors (VIP ~ 2.14 at most) with negative coefficients. Similar to that shown in Figures 4 and 5, we investigated the relationship between the dielectric constants and the important DOS spectral data. Figure 6 shows the relationship obtained using partial DOS for the A-cations (in the 3.8–4.4 eV range). The materials with high dielectric constants do not have a partial DOS for the A ions in the 3.8–4.4 eV energy range, whereas Mg (red), Zn (magenta), and Ca (orange)-containing compounds with low dielectric constants are more likely to show a relatively high partial DOS. Hence, the descriptors derived from the DOS for the A ions can be used to screen materials with low dielectric constants. The partial DOS for the B-cations (in the 1.9–3.9 eV range) were also extracted as one of the better descriptors (VIP ~ 2.63 at most) with positive coefficients. Interestingly, the data points can be clearly separated into two areas with a border at 10.0 of the horizontal value, as shown in Figure 7: one for the Ti-containing materials (red data points) and the other for the other materials. This figure shows that the B-cation DOS descriptor plays a significant role in improving the prediction of the dielectric constants of the Ti-containing materials. As mentioned above (factor (b)), the inclusion of Ti at the perovskite B-site leads to a higher dielectric constant, and the partial DOS for the B-ions is a good descriptor to distinguish the Ti ions from the other B ions. Thus, we infer that the descriptor also shows high VIP scores.

Overall, the six descriptors extracted from our PLS regression analysis play important roles in predicting the dielectric constants from the VIP analysis. However, a single descriptor cannot directly express the dielectric properties; therefore any correlation between a single descriptor and the dielectric constant is poor. No strong correlations exist between the dielectric constant and the two descriptors, although we investigated the relationships between dielectric constant and two descriptors among the six explanatory variables a − f (see Supplemental Figure S8). Thus, we conclude that multiple factors complementarily affect the dielectric performance.

## 4. Conclusions

In this study, descriptors for predicting the dielectric constants of perovskite-type oxides were investigated using first-principles calculations and PLS regression analysis. The basic physical and chemical characteristics, such as the DOS spectra and atomic charge, were set as explanatory variables in the PLS regression model. The PLS regression model showed a high accuracy in predicting the dielectric constants of the perovskite-type oxides. In addition, we confirmed that six explanatory variables, namely the Shannon ionic radii of the A cations, differences between the Bader and formal charges of the B cations, the RDF spectra for the A − A and B − B combinations, and the DOS spectra of the A and B cations in the conduction bands, strongly affect the dielectric constants of the perovskite-type oxides in the PLS regression analysis. In particular, the charge difference (ionicity) of the B cations and the DOS spectral data for the conduction bands of the B cations showing high VIP scores were extracted as better descriptors for predicting the dielectric constants. The informatics-aided approach can be used to build a multivariate regression model to predict a particular property from physical and chemical properties of the target materials and thus provide important explanatory variables for the regression model. This approach is extensible to other compositional series. For example, the PLS regression results show comparable quality of fit even for the datasets that include perovskite samples that contain lone electron pairs, Sn^2+^, and Pb^2+^ at their A-sites (see Supplemental Figure S9).

## Supplementary Material

Supplemental Material

## References

[1] Butler KT, Frost JM, Skelton JM, et al. Computational materials design of crystalline solids. Chem Soc Rev. 2016;45:6138–6146. 26992173 10.1039/c5cs00841g PMC5103860

[2] Armiento R, Kozinsky B, Fornari M, et al. Screening for high-performance piezoelectrics using high-throughput density functional theory. Phys Rev B. 2011;84:014103.

[3] Fujimura K, Seko A, Koyama Y, et al. Accelerated materials design of lithium superionic conductors based on first-principles calculations and machine learning algorithms. Adv Energy Mater. 2013;3:980–985.

[4] Jalem R, Nakayama M, Kasuga T. An efficient rule-based screening approach for discovering fast lithium ion conductors using density functional theory and artificial neural networks. J Mater Chem A. 2014;2:720–734.

[5] Toher C, Plata JJ, Levy O, et al. High-throughput computational screening of thermal conductivity, debye temperature, and grüneisen parameter using a quasiharmonic debye model. Phys Rev B. 2014;90:174107.

[6] Jalem R, Kimura M, Nakayama M, et al. Informatics-aided density functional theory study on the li ion trasport of tavorite-type LiMTO_4_F (M^3+^−T^5+^, M^2+^−T^6+^). J Chem Inf Model. 2015;55:1158–1168. 26000780 10.1021/ci500752n

[7] Hinuma Y, Hatakeyama T, Kumagai Y, et al. Discovery of earth-abundant nitride semiconductors by computational screening and high-pressure synthesis. Nat Commun. 2016;7:11962. 27325228 10.1038/ncomms11962 PMC4919542

[8] Pankajakshan P, Sanyal S, de Noord OE, et al. Machine learning and statistical analysis for materials science: stability and transferability of fingerprint descriptors and chemical insights. Chem Mater. 2017;29:4190–4201.

[9] Davies DW, Butler KT, Skelton JM, et al. Computer-aided design of metal chalcohalide semiconductors: from chemical composition to crystal structure. Chem Sci. 2018;9:1022–1030. 29675149 10.1039/c7sc03961a PMC5883896

[10] Katritzky AR, Gordeeva EV. Traditional topological indices vs electronic, geometrical, and combined molecular descriptors in QSAR/QSPR Research. J Chem Inf Comput Sci. 1993;33:835–857. 8113335 10.1021/ci00016a005

[11] de Lima Ribeiro FA, Ferreira MMC. QSPR models of boiling point, octanol−water partition coefficient and retention time index of polycyclic aromatic hydrocarbons. J Mol Struct (Theochem). 2003;366:109–126.

[12] Yang S, Lu W, Chen N, et al. Support vector regression based QSPR for the prediction of some physicochemical properties of alkyl benzenes. J Mol Struct (Theochem). 2005;719:119–127.

[13] Hernández N, Kiralj R, Ferreira MMC, et al. Critical comparative analysis, validation and interpretation of svm and pls regression models in a QSPR Study on HIV-1 protease inhibitors. Chemom Intell Lab Stst. 2009;98:65–77.

[14] Seko A, Maekawa T, Tsuda K, et al. Machine learning with systematic density-functional theory calculations: application to melting temperatures of single- and binary-component solids. Phys Rev B. 2014;89:054303.

[15] Jain A, Hautier G, Ong SP, et al. New opportunities for materials informatics: resources and data mining techniques for uncovering hidden relationships. J Mater Res. 2016;31:977–994.

[16] Seko A, Hayashi H, Nakayama K, et al. Representation of compounds for machine-learning prediction of physical properties. Phys Rev B. 2017;95:144110.

[17] Takahashi K, Tanaka Y. Unveiling descriptors for predicting the bulk modulus of amorphous carbon. Phys Rev B. 2017;95:054110.

[18] Yamaguchi S, Nishimura T, Hibe Y, et al. Regularized regression analysis of digitized molecular structures in organic reactions for quantification of steric effects. J Comput Chem. 2017;38:1825–1833. 28349554 10.1002/jcc.24791

[19] Liu Y, Zhao T, Ju W, et al. Design using machine learning. J Materiomics. 2017;3:159–177.

[20] Broderick SR, Rajan K. Eigenvalue decomposition of spectral features in density of states curves. Europhys Lett. 2011;95:57005.

[21] Broderick SR, Aourag H, Rahan K. Data mining density of states spectra for crystal structure classification: an inverse problem approach. Stat Anal Data Min. 2009;1:353–360.

[22] Broderick SR, Aourag H, Rajan K. Classification of oxide compounds through data-mining density of states spectra. J Am Ceram Soc. 2011;94:2974–2980.

[23] Broderick SR, Rajan K. Information science for materials discovery and design. Lookman T, Alexander FJ, Rajan K, editors. Springer International Publishing, Switzerland: 2016.

[24] Barrett JH. Dielectric constant in perovskite type crystals. Phys Rev. 1952;86:118–120.

[25] King-Smith RD, Vanderbilt D. First-principles investigation of ferroelectricity in perovskite compounds. Phys Rev B. 1994;49:5828–5844. 10.1103/physrevb.49.5828 10011559

[26] Ph. Ghosez J, Michenaud P, Gonze X. Dynamical atomic charges: the case of ABO_3_ compounds. Phys Rev. 1998;58:6224–6240.

[27] van Benthem K, Elsässer C, French RH. Bulk electronic structure of SrTiO_3_: experiment and theory. J Appl Phys. 2001;90:6156–6164.

[28] Hohenberg P, Kohn W. Inhomogeneous electron gas. Phys Rev. 1964;136:B864–B870.

[29] Geladi P, Kowalski BR. Partial least-squares regression: a tutorial. Anal Chim Acta. 1986;185:1–17.

[30] Wold S, Sjöström M, Eriksson L. PLS-regression: a basic tool of chemometrics. Chemom Intell Lab Syst. 2001;58:109–130.

[31] Sawatsky ML, Clyde M, Meek F. Partial least squares regression in the social sciences. Tutor Quant Methods Psychol. 2015;11:52–62.

[32] Kresse G, Hafner J. Ab initio molecular dynamics for liquid metals. Phys Rev B. 1993;47:558–561. 10.1103/physrevb.47.558 10004490

[33] Kresse G, Hafner J. Ab initio molecular-dynamics simulation of the liquid-metal–amorphous-semiconductor transition in germanium. Phys Rev B. 1994;49:14251–14269. 10.1103/physrevb.49.14251 10010505

[34] Kresse G, Furthmüller J. Efficiency of ab-initio total energy calculations for metals and semiconductors using a plane-wave basis set. Comput Mater Sci. 1996;6:15–50. 10.1103/physrevb.54.11169 9984901

[35] Kresse G, Furthmüller J. Efficient iterative schemes for ab initio total-energy calculations using a plane-wave basis set. Phys Rev B. 1996;54:11169–11186. 10.1103/physrevb.54.11169 9984901

[36] Blöchl PE. Projector augmented-wave method. Phys Rev B. 1994;50:17953–17979. 10.1103/physrevb.50.17953 9976227

[37] Kresse G, Joubert D. From ultrasoft pseudopotentials to the projector augmented-wave method. Phys Rev B. 1999;59:1758–1775.

[38] Perdew JP, Ruzsinszky A, Csonka GI, et al. Restoring the density-gradient expansion for exchange in solids and surfaces. Phys Rev Lett. 2008;100:136406−1-4. 10.1103/PhysRevLett.100.136406 18517979

[39] Pauling L. The nature of the chemical bond. 3rd ed. Ithaca (NY): Cornell University Press; 1960.

[40] Allred AL. Electronegativity values from thermochemical data. J Inorg Nucl Chem. 1961;17:215–221.

[41] Shannon RD. Revised effective ionic radii and systematic studies of interatomic distances in halides and chalcogenides. Acta Crystallogr Sect A. 1976;32:751–767.

[42] Tang W, Sanville E, Henkelman G. A grid-based bader analysis algorithm without lattice bias. J Phys Condens Matter: Inst Phys J. 2009;21:084204. 10.1088/0953-8984/21/8/084204 21817356

[43] Murnaghan FD. The compressibility of media under extreme pressures. Proc Nat Acad Sci. 1944;30:244–247. 16588651 10.1073/pnas.30.9.244 PMC1078704

[44] Baroni S, Giannozzi P, Testa A. Green’s-function approach to linear response in solids. Phys Rev Lett. 1987;58:1861–1864. 10034557 10.1103/PhysRevLett.58.1861

[45] Gajdoš M, Hummer K, Kresse G, et al. Linear optical properties in the projector-augmented wave methodology. Phys Rev B. 2006;73:045112.

[46] Wold S, Johansson A, Cochi M. 3D QSAR in drug design, theory, methods, and applications. Kubinyi H, editors. Leiden: ESCOM Science Publishers; 1993.

[47] Farrés M, Platikanov S, Tsakovski S, et al. Comparison of the Variable Importance in Projection (VIP) and of the Selectivity Ratio (SR) methods for variable selection and interpretation. J Chemom. 2015;29:528–536.

[48] Cox I, Gaudard M. Discovering partial least squares with JMP®. North Carolina (USA): SAS Institute Inc., Cary; 2013.

[49] JMP® Pro 11. North Carolina (USA): SAS Institute Inc., Cary; 2013.

[50] Lemanov VV, Sotnikov AV, Smirnova EP, et al. Perovskite CaTiO_3_ as an incipient ferroelectric. Solid State Commun. 1999;110:611–614.

[51] Levin I, Amos TG, Bell SM, et al. Dielectric anomaly in the BaZrO_3_–CaZrO_3_ system. J Solid State Chem. 2003;175:170–181.

[52] Müller KA, Burkard H. SrTiO_3_: an intrinsic quantum paraelectric below 4 K. Phys Rev B. 1979;19:3593–3602.

[53] Parida S, Rout SK, Subramanian V, et al. Structural, microwave dielectric properties and dielectric resonator antenna studies of Sr(Zr*_x_*Ti_1−*x*_)O_3_ ceramics. J Alloys Compd. 2012;528:126–134.

[54] He L, Liu F, Hautier G, et al. Accuracy of generalized gradient approximation functionals for density-functional perturbation theory calculations. Phys Rev B. 1999;89:064305.

[55] Yim K, Yong Y, Lee J, et al. Novel high-κ dielectrics for next-generation electronic devices screened by automated ab initio calculations. NPG Asia Mater. 2015;7:e190.

[56] Stokes HT, Hatch DM. FINDSYM: program for identifying the space-group symmetry of a crystal. J Appl Cryst. 2005;38:237–238.

