# Supplementary material for: Descriptors for dielectric constants of perovskite-type oxides by materials informatics with first-principles density functional theory
Source: Sci Technol Adv Mater. 2020 Feb 25;21(1):92–9. doi: 10.1080/14686996.2020.1724824 (PMC7054915; doi:10.1080/14686996.2020.1724824)
Supplement: Supplemental Material [file TSTA_A_1724824_SM8120.pdf]

Supplemental Material for

Descriptors for dielectric constants of perovskite-type oxides by materials informatics with first-principles density functional theory

Yusuke Noda[a], Masanari Otake[b] and Masanobu Nakayama[a,b,c,d]

[a]*Center for Materials research by Information Integration (CMI[2]), Research and Services Division of Materials Data and Integrated System (MaDIS), National Institute for Materials Science (NIMS), 1-2-1 Sengen, Tsukuba, Ibaraki 305-0047, Japan*

[b]*Frontier Research Institute for Materials Science (FRIMS), Nagoya Institute of Technology, Gokiso, Showa, Nagoya, Aichi 466-8555, Japan*

[c]*Global Research Center for Environment and Energy based on Nanomaterials Science (GREEN), National Institute for Materials Science (NIMS), 1-1 Namiki, Tsukuba, Ibaraki 305-0047, Japan*

[d]*Elements Strategy Initiative for Catalysts and Batteries (ESICB), Kyoto University, 1-30 Goryo-Ohara, Nishikyo, Kyoto 615-8245, Japan*

Supplemental Section 1. Underlying model for partial least-squares regression

Partial least-squares (PLS) regression is a useful multivariate regression analysis method. Consider linear relationships of n samples between explanatory variables x_{ij} ($i = 1{-}n$; $j = 1{-}m$) and objective variables y_i (in this paper, only a single variable y_i is considered):

$$y_i = b_1x_{i1} + b_2x_{i2} + b_3x_{i3} + \ldots + b_mx_{im} + f_i \quad \text{(S1)},$$

where b_j and f_i are the coefficients of x_{ij} and the residual, respectively. Eq. S1 can be rewritten using objective variables $\boldsymbol{y} = (y_1\ y_2\ y_3\ \ldots\ y_n)^{\mathrm{T}}$, regression coefficients $\boldsymbol{b} = (b_1\ b_2\ b_3\ \ldots\ b_m)^{\mathrm{T}}$, $\boldsymbol{y}$-residuals $\boldsymbol{f} = (f_1\ f_2\ f_3\ \ldots\ f_n)^{\mathrm{T}}$, and explanatory variables $\boldsymbol{X} = (\boldsymbol{x}_1\ \boldsymbol{x}_2\ \boldsymbol{x}_3\ \ldots\ \boldsymbol{x}_m)$:

$$\boldsymbol{y} = \boldsymbol{X}\boldsymbol{b} + \boldsymbol{f} \quad \text{(S2)},$$

where $\boldsymbol{x}_j = (x_{1j}\ x_{2j}\ x_{3j}\ \ldots\ x_{nj})^{\mathrm{T}}$. In this study, we use the nonlinear iterative partial least squares (NIPALS) [S1] algorithm to predict the regression coefficients between $\boldsymbol{X}$ and $\boldsymbol{y}$, which are scaled by a combination of mean centering and standardization (in our

case, the NIPALS algorithm is non-iterative because the objective variables $\boldsymbol{y}$ are not expressed as a matrix but rather a single vector). The objective variables $\boldsymbol{y}$ can be expressed as follows in the NIPALS algorithm:

$$\boldsymbol{y} = \boldsymbol{Tc} + \boldsymbol{f} = \boldsymbol{XW}(\boldsymbol{P}^{\mathrm{T}}\boldsymbol{W})^{-1}\boldsymbol{c} + \boldsymbol{f} \qquad \text{(S3)},$$

where $\boldsymbol{T} = (\boldsymbol{t}_1\ \boldsymbol{t}_2\ \boldsymbol{t}_3\ ...\ \boldsymbol{t}_a)$, ($a$ indicates the number of factors considered in the PLS regression), $\boldsymbol{P} = (\boldsymbol{p}_1\ \boldsymbol{p}_2\ \boldsymbol{p}_3\ ...\ \boldsymbol{p}_a)$, $\boldsymbol{W} = (\boldsymbol{w}_1\ \boldsymbol{w}_2\ \boldsymbol{w}_3\ ...\ \boldsymbol{w}_a)$, and $\boldsymbol{c} = (c_1\ c_2\ c_3\ ...\ c_a)^{\mathrm{T}}$ are $\boldsymbol{X}$-scores, $\boldsymbol{X}$-loadings, $\boldsymbol{X}$-weights, and $\boldsymbol{y}$-weights, respectively. The first $\boldsymbol{X}$-weight component $\boldsymbol{w}_1$ and the first $\boldsymbol{X}$-score component $\boldsymbol{t}_1$ are calculated as follows:

$$\boldsymbol{w}_1 = \boldsymbol{X}^{\mathrm{T}}\boldsymbol{y}/||\boldsymbol{X}^{\mathrm{T}}\boldsymbol{y}|| \qquad \text{(S4)},$$
$$\boldsymbol{t}_1 = \boldsymbol{X}\boldsymbol{w}_1 \qquad \text{(S5)}.$$

Component $\boldsymbol{w}_a$ is obtained in order to maximize covariance between $\boldsymbol{X}$ and $\boldsymbol{y}$. The component $\boldsymbol{t}_a$ is the latent variable used to build the multivariate regression model with a small number of variables. The first $\boldsymbol{X}$-loading component $\boldsymbol{p}_1$ and the first $\boldsymbol{y}$-weight component c_1 can be obtained by a single regression to $\boldsymbol{X}$ and $\boldsymbol{y}$ using the latent variable $\boldsymbol{t}_1$. Therefore, the components $\boldsymbol{p}_1$ and c_1 are also calculated as follows:

$$\boldsymbol{p}_1 = \boldsymbol{X}^{\mathrm{T}}\boldsymbol{t}_1/\boldsymbol{t}_1^{\mathrm{T}}\boldsymbol{t}_1 \qquad \text{(S6)},$$
$$c_1 = \boldsymbol{y}^{\mathrm{T}}\boldsymbol{t}_1/\boldsymbol{t}_1^{\mathrm{T}}\boldsymbol{t}_1 \qquad \text{(S7)}.$$

Finally, the projection data to $\boldsymbol{X}$ and $\boldsymbol{y}$ (i.e., $\boldsymbol{t}_1\boldsymbol{p}_1^{\mathrm{T}}$ and $\boldsymbol{t}_1 c_1$) are removed from the original $\boldsymbol{X}$ and $\boldsymbol{y}$:

$$\boldsymbol{X}' = \boldsymbol{X} - \boldsymbol{t}_1\boldsymbol{p}_1^{\mathrm{T}} \qquad \text{(S8)},$$
$$\boldsymbol{y}' = \boldsymbol{y} - \boldsymbol{t}_1 c_1 \qquad \text{(S9)}.$$

The deflated data $\boldsymbol{X}'$ and $\boldsymbol{y}'$ are used as the next $\boldsymbol{X}$ and $\boldsymbol{y}$ in Eq. S4 to determine the second components $\boldsymbol{t}_2$, $\boldsymbol{p}_2$, and c_2. The latent variables $\boldsymbol{t}_a$ are estimated so that they are uncorrelated with each other by repeating the deflation execution to $\boldsymbol{X}$ and $\boldsymbol{y}$. After calculating the a-th principal components of each variable, we can obtain the PLS regression coefficients $\boldsymbol{b}^{\mathrm{PLS}}$ with predicted objective variables $\hat{\boldsymbol{y}}$ that satisfy $\hat{\boldsymbol{y}} = \boldsymbol{X}\boldsymbol{b}^{\mathrm{PLS}}$:

$$\boldsymbol{b}^{\mathrm{PLS}} = \boldsymbol{W}(\boldsymbol{P}^{\mathrm{T}}\boldsymbol{W})^{-1}\boldsymbol{c} \qquad \text{(S10)}.$$

In the PLS method, we use an index to determine the superiority of the regression coefficients. Wold et al. proposed variable importance in projection (VIP) scores,

which evaluate the influence of explanatory variables $\boldsymbol{X}$ on the PLS regression model [S2, S3]. The VIP score for the j-th variable is expressed as:

$$\mathrm{VIP}_j = \sqrt{\frac{\sum_f^F w_{jf}^2 \cdot \mathrm{SSY}_f \cdot J}{\mathrm{SSY}_{\mathrm{total}} \cdot F}} \quad \text{(S11).}$$

In this equation, w_{jf} indicates the weights of the j-th variable and f-th component. SSY_f is the sum of the squares of $\boldsymbol{y}$ explained by the f-th component and $\mathrm{SSY}_{\mathrm{total}}$ is the total sum of the squares of $\boldsymbol{y}$ explained by all components; both values are given as follows:

$$\mathrm{SSY}_f = (b^{\mathrm{PLS}}{}_f)^2 \boldsymbol{t}_f^{\mathrm{T}} \boldsymbol{t}_f \quad \text{(S12),}$$

$$\mathrm{SSY}_{\mathrm{total}} = (\boldsymbol{b}^{\mathrm{PLS}})^2 \boldsymbol{T}^{\mathrm{T}} \boldsymbol{T} \quad \text{(S13),}$$

where $b^{\mathrm{PLS}}{}_f$ is the f-th component of the PLS regression coefficients $\boldsymbol{b}^{\mathrm{PLS}}$, J is the number of explanatory variables $\boldsymbol{X}$, and F is the total number of components. Explanatory variables with larger VIP scores are important for building the PLS regression model.

Supplemental Section 2. Additional PLS regression analysis

Additional PLS regressions were performed to investigate (i) DOS energy alignment and (ii) the inclusion of perovskites that contains Pb^{2+} and Sn^{2+} at A-sites. Due to the lack of bulk modulus data for Pb^{2+}- and Sn^{2+}-containing perovskites and data format alignment, we recalculated the entire PLS regression using slightly different datasets of explanatory variables from those shown in Figure 2 in the main text. Therefore, the diagnostic plots shown in Supplemental Figures S4(a) and S9(a), which correspond to the datasets in Figure 2 in main text, shows differences that help to compare the PLS regression results under the same regression conditions.

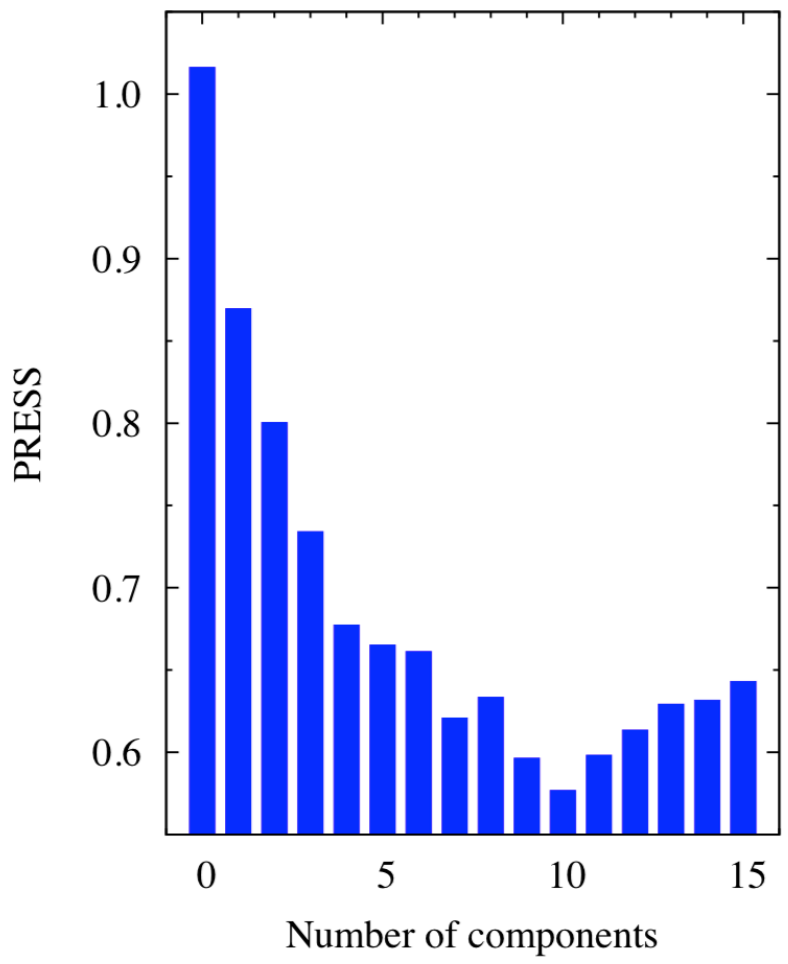

Supplemental Figure S1. Plot of predicted residual error sum of squares (PRESS) against the number of components in the PLS regression model for test data. The minimum PRESS value occurs at ten components.

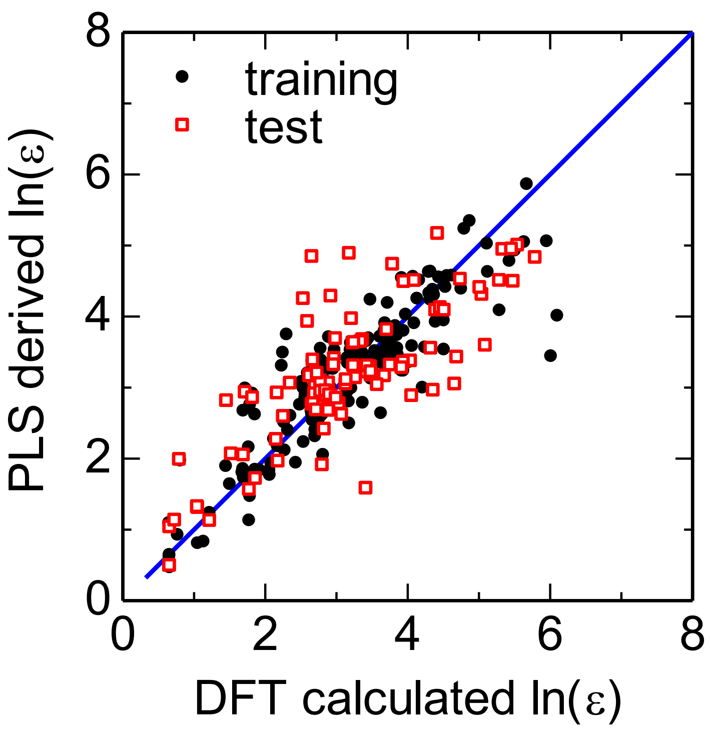

Supplemental Figure S2. Diagnostic PLS-regression plots of logarithmic dielectric constant, ln (ε), for the samples only perovskite with vertices-shared BO_6 octahedra, *i.e.* face- and edge-shared perovskites of $R\bar{3}$ and $P6_3/mmc$ symmetries are removed from the samples. The resulting statistical evaluation parameters are as follows; RMSE(training) = 0.43, RMSE(test) = 0.57, R^2(training) = 0.79, and R^2(test) = 0.60.

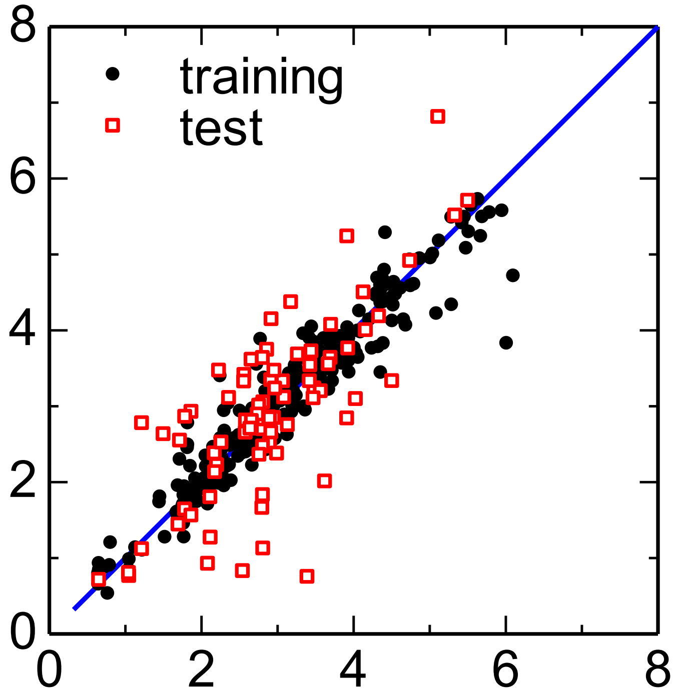

Supplemental Figure S3. Diagnostic PLS-regression plots of logarithmic dielectric constant, ln (ε), obtained by adding band gap data to the explanatory variables. The resulting statistical evaluation parameters are as follows; RMSE(training) = 0.29, RMSE(test) = 0.54, R^2(training) = 0.92, and R^2(test) = 0.84.

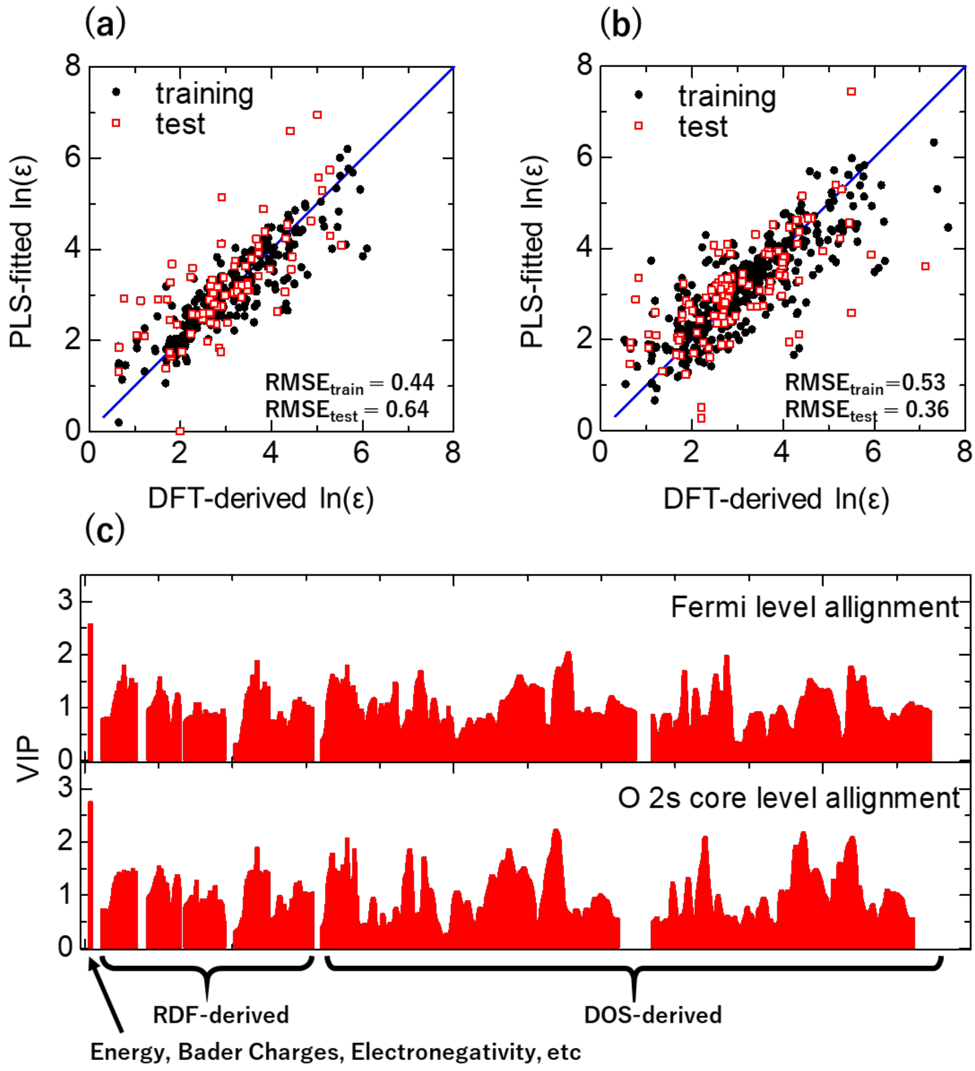

Supplemental Figure S4. Diagnostic PLS-regression plots of logarithmic dielectric constant, ln (ε), using the DOS energy scale aligned with: (a) the Fermi level and (b) the O 2s core level for DOS-derived explanatory variables. RMSEs of the training and test data are presented in the plots. (c) VIPs for various descriptors derived from PLS regression. Upper and lower bar graphs correspond to PLS derived VIPs using DOS descriptors whose energies are aligned to the Fermi level and the O 2s core level, respectively.

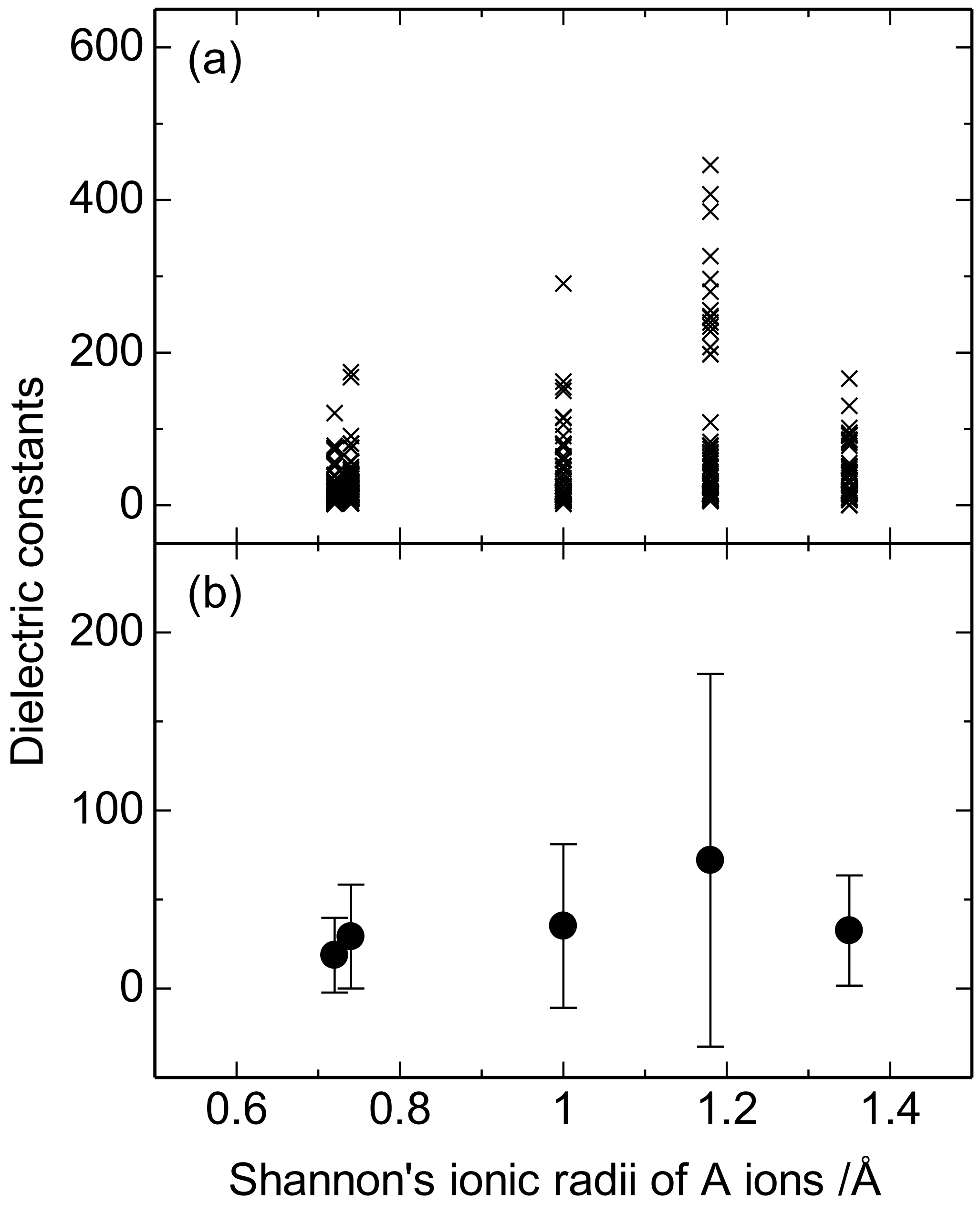

Supplemental Figure S5. (a) Relationship between DFPT-derived dielectric constants and Shannon’s ionic radii of A ions, which has relatively high PLS-VIP scores. (b) Averaged dielectric constant (dot) and corresponding standard deviation (error bar) for each A ion.

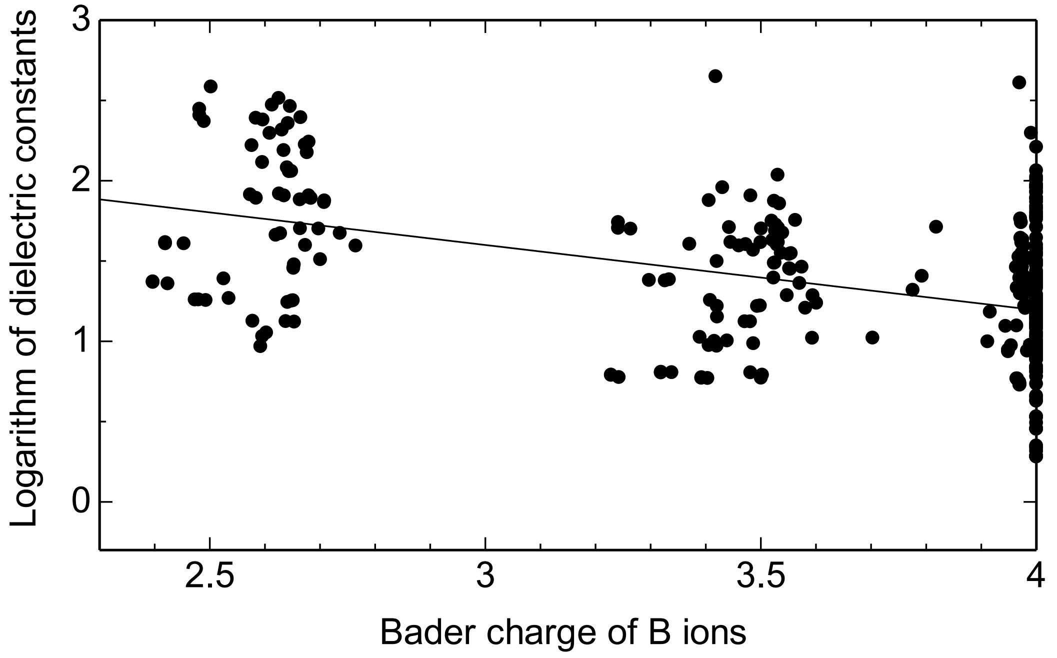

Supplemental Figure S6. Relationship between Bader charge of B ions and dielectric constants. (Several compounds with a low dielectric constant ($\varepsilon < 1$) are removed from the figure as outliers.) The line in the figure corresponds to the results of the least-squares linear fitting. The correlation coefficient R is –0.45, and the root-mean-square error (RMSE) is 0.40 for the fitted function.

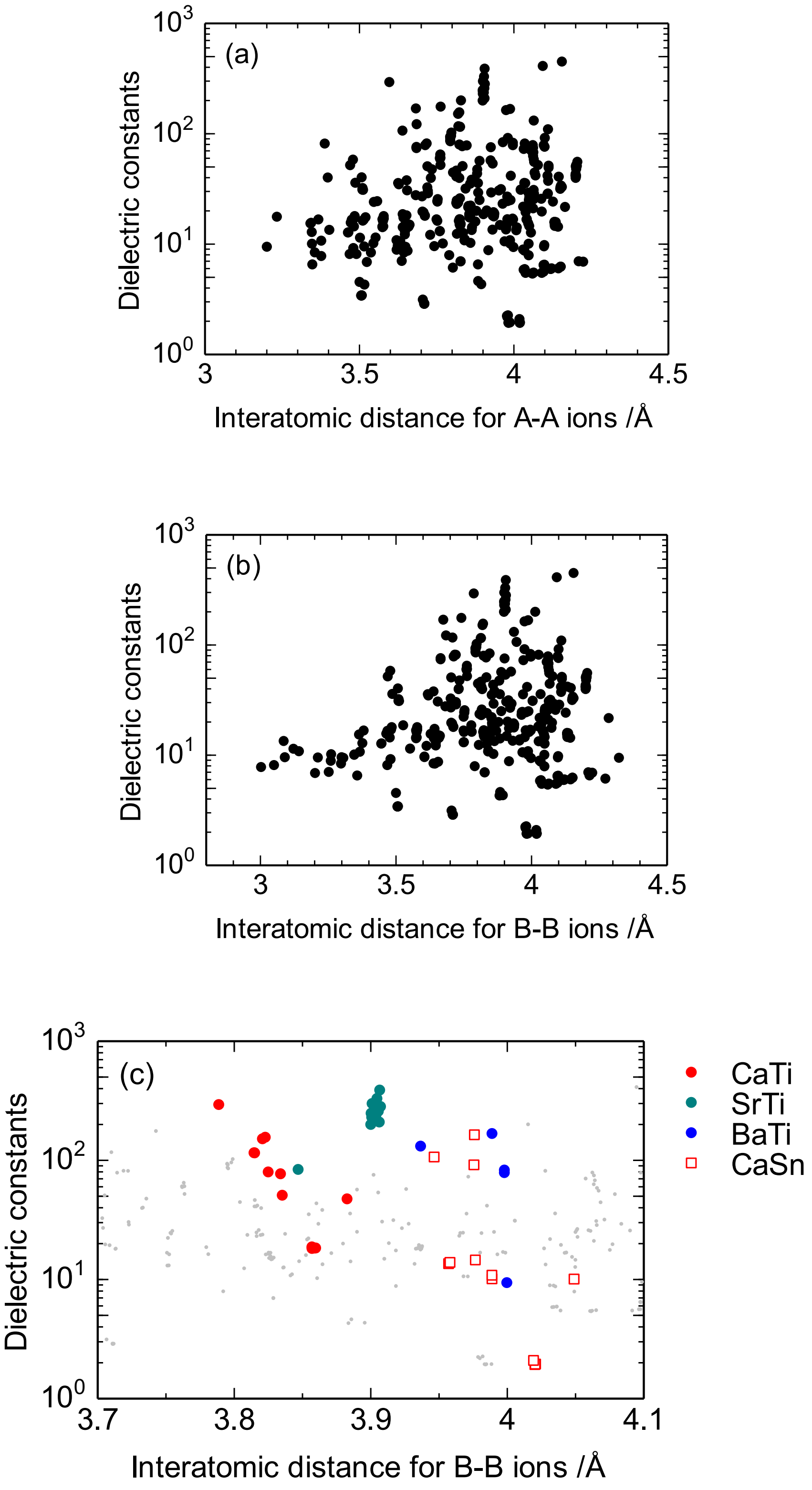

Supplemental Figure S7. Relationship between dielectric constants and interatomic distance between (a) two A sites and (b) two B sites. Panel (c) is a magnification of panel (b) for several specific A and B compositions.

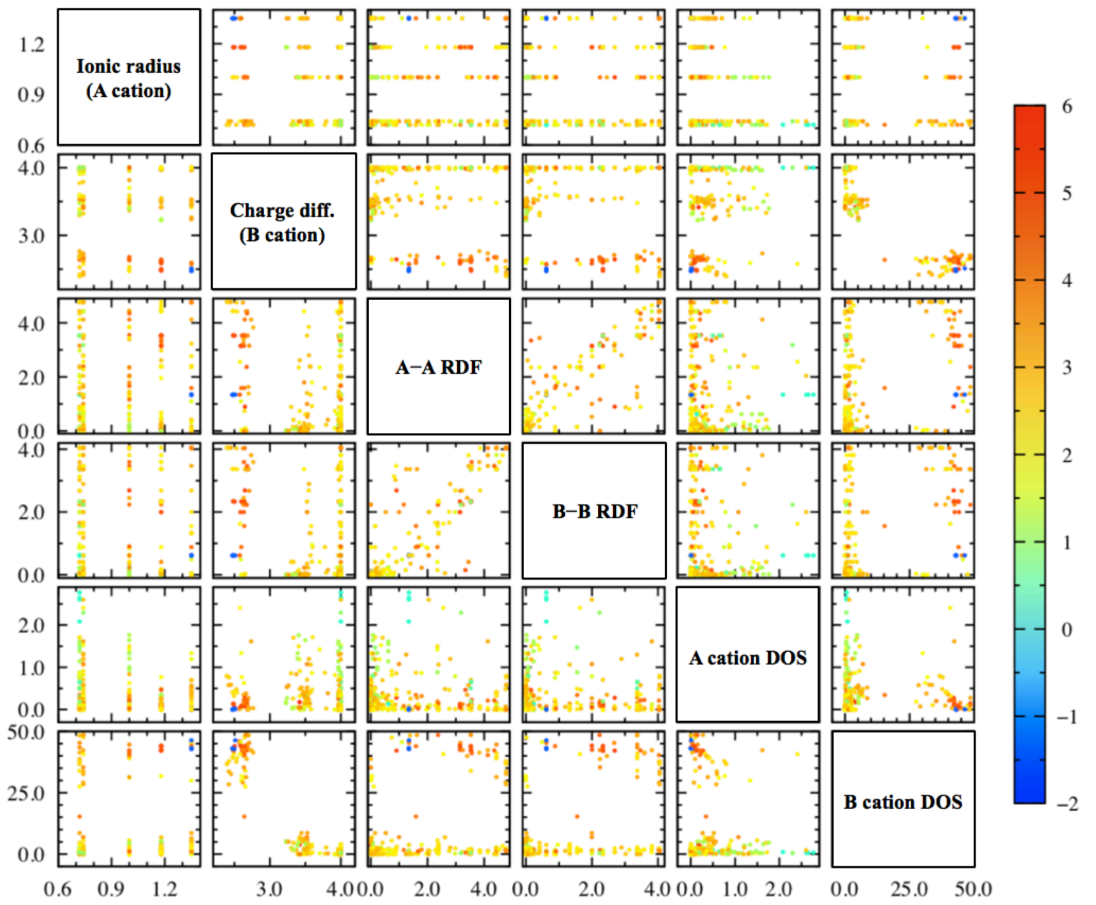

Supplemental Figure S8. Correlation matrix of dielectric constants as functions of two descriptors among the six explanatory variables a−f for all sample data. The dielectric constants (ln ε) are shown by color gradation. Relatively high dielectric materials (red plots) are condensed in some of regions, but the blue plots also coexist in the same region, indicating no significant correlation (one of examples is the correlation graph between B cation DOS and charge difference).

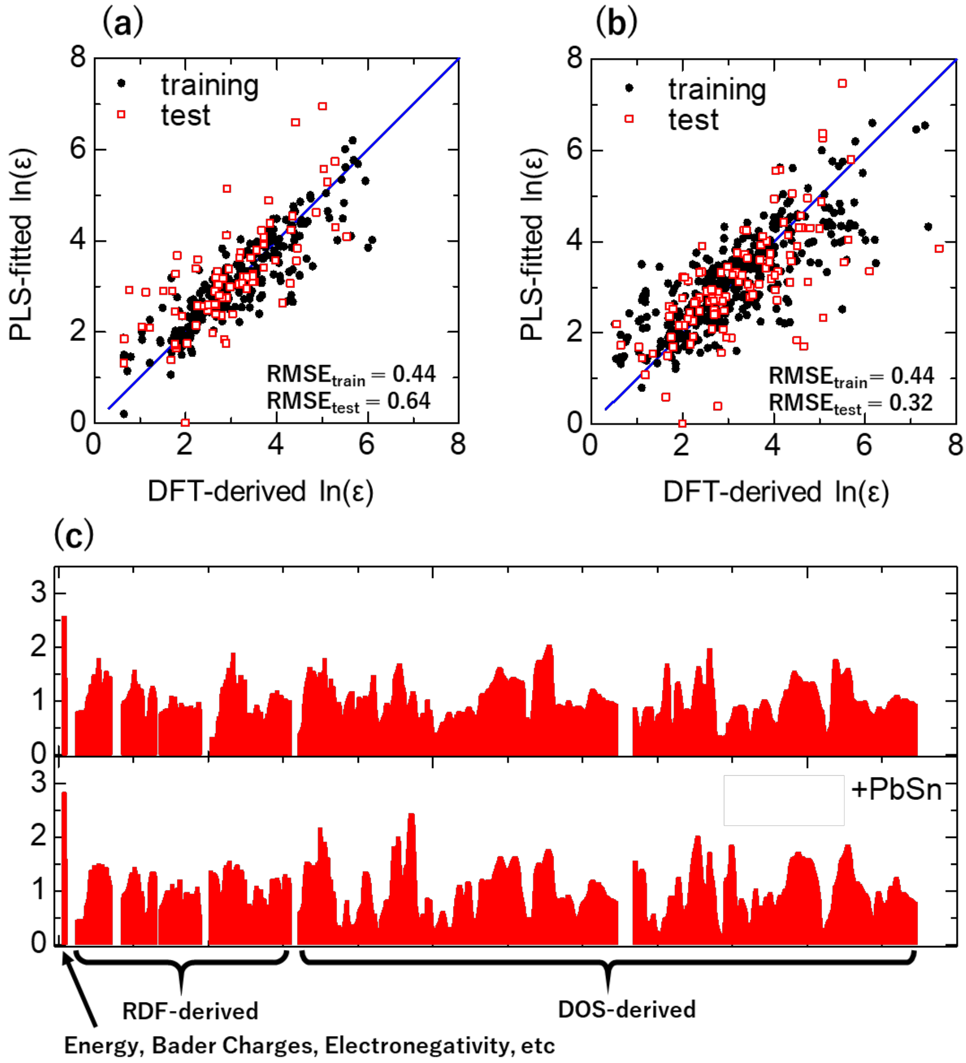

Supplemental Figure S9. Diagnostic PLS-regression plots of logarithmic dielectric constant, ln (ε) for the sample sets (a) without and (b) with perovskites that contains Pb^{2+} or Sn^{2+} at A-site. RMSEs of training and test data are presented in the plots. (c) VIPs for various descriptors derived from PLS regression. Upper and lower bar graphs correspond to PLS derived VIPs with and without perovskites that contains Pb^{2+} or Sn^{2+} at A-site in the regression samples, respectively.

References

[S1] S. Wold, M. Sjöström, and L. Eriksson, PLS-Regression: A Basic Tool of Chemometrics, *Chemom. Intell. Lab. Syst.* **58**, 109-130 (2001).

[S2] S. Wold, A. Johansson, and M. Cochi, in *3D QSAR in Drug Design, Theory, Methods, and Applications*, edited by H. Kubinyi (ESCOM Science Publishers, Leiden, 1993).

[S3] M. Farrés, S. Platikanov, S. Tsakovski, and R. Tauler, Comparison of the Variable Importance in Projection (VIP) and of the Selectivity Ratio (SR) Methods for Variable Selection and Interpretation, *J. Chemom.* **29**, 528-536 (2015).
